# Revealing hidden states in visual working memory using electroencephalography

**DOI:** 10.3389/fnsys.2015.00123

**Published:** 2015-09-03

**Authors:** Michael J. Wolff, Jacqueline Ding, Nicholas E. Myers, Mark G. Stokes

**Affiliations:** ^1^Department of Experimental Psychology, University of GroningenGroningen, Netherlands; ^2^Oxford Centre for Human Brain Activity, University of OxfordOxford, UK; ^3^Department of Experimental Psychology, University of OxfordOxford, UK

**Keywords:** EEG, multivariate pattern analysis, dynamic coding, hidden state, visual working memory

## Abstract

It is often assumed that information in visual working memory (vWM) is maintained via persistent activity. However, recent evidence indicates that information in vWM could be maintained in an effectively “activity-silent” neural state. Silent vWM is consistent with recent cognitive and neural models, but poses an important experimental problem: how can we study these silent states using conventional measures of brain activity? We propose a novel approach that is analogous to echolocation: using a high-contrast visual stimulus, it may be possible to drive brain activity during vWM maintenance and measure the vWM-dependent impulse response. We recorded electroencephalography (EEG) while participants performed a vWM task in which a randomly oriented grating was remembered. Crucially, a high-contrast, task-irrelevant stimulus was shown in the maintenance period in half of the trials. The electrophysiological response from posterior channels was used to decode the orientations of the gratings. While orientations could be decoded during and shortly after stimulus presentation, decoding accuracy dropped back close to baseline in the delay. However, the visual evoked response from the task-irrelevant stimulus resulted in a clear re-emergence in decodability. This result provides important proof-of-concept for a promising and relatively simple approach to decode “activity-silent” vWM content using non-invasive EEG.

## Introduction

Visual Working memory (vWM) is essential for high-level cognition. By keeping task-relevant information in mind, vWM provides a functional basis for complex behaviors based on time-extended goals and contextual contingencies. Some of the most influential models of vWM are built on the intuitive notion that maintenance is directly related to the persistence of stationary activity states, representing specific content in vWM from the moment of encoding until that content is needed for behavior (Goldman-Rakic, 1995; Curtis and D'Esposito, 2003). Persistent activity models have obvious appeal—vWM effectively preserves a freeze-frame snapshot of past experience until it is no longer required. However, there are gaps in the argument for persistent activity models of vWM.

Accumulating evidence suggests that vWM is not always accompanied by persistent delay activity (Sreenivasan et al., 2014). For example, a recent study in non-human primates showed that content-specific delay activity can be effectively abolished during dual task interference, even though vWM-guided behavior is relatively spared (Watanabe and Funahashi, 2014). Robust delay activity returned when attention was refocused on the vWM-task. Similarly, human studies using non-invasive brain imaging suggest that activity patterns during maintenance delays correspond only to attended items (Lewis-Peacock et al., 2011). Unattended items do not seem to have a corresponding activity state, even though such unattended items are still maintained in vWM (Olivers et al., 2011; Larocque et al., 2014). As in the non-human primate study, the activity state of unattended items becomes apparent once attention is directed to them (Lewis-Peacock et al., 2011; Lewis-Peacock and Postle, 2012).

These results suggest that delay activity is not strictly necessary for maintenance in vWM. Dissociating vWM-performance from persistent delay activity implies that some form of “activity-silent” neural state contributes to maintenance in vWM (Stokes, 2015). For example, a synaptic model of vWM proposes that information is encoded in item-specific patterns of functional connectivity (Mongillo et al., 2008; Sugase-Miyamoto et al., 2008). Essentially, activity patterns during encoding drive content-specific changes in short-term synaptic plasticity (Zucker and Regehr, 2002). Although the temporary synaptic trace is effectively “activity silent,” this hidden neural state can be read out from the network during processing of a memory probe. Mongillo et al. (2008) focused on known mechanisms of short-term synaptic plasticity; however, other neurophysiological factors could also pattern hidden states for vWM-guided behavior (Buonomano and Maass, 2009). The key principle is that activity-dependent changes in the hidden neural state could be important for maintaining information in vWM.

One reason that persistent-activity models of vWM have been so pervasive in the past is that it is much easier to find confirmatory evidence with conventional measures, such as elevated delay-period firing (Fuster and Alexander, 1971) or pattern decoding during the delay period (Harrison and Tong, 2009). Disconfirmatory evidence is essentially a null effect. Therefore, to evaluate the possible contributions of hidden states to vWM maintenance, it is necessary to develop measures that are capable of revealing them. Previously, we found that a neutral task-irrelevant stimulus presented during a vWM delay period generated vWM-specific patterns of activity in monkey prefrontal cortex (PFC; Stokes et al., 2013). We suggested that this context-dependent response pattern could reflect differences in hidden state. For illustration, consider echolocation (e.g., sonar), where a simple impulse (e.g., “ping”) is used to probe hidden contours of unseen structure. Analogously, the impulse response to neural perturbation should co-depend on the pattern of input activity and the hidden state of the network. If the input pattern is held constant, we can attribute differences in the output to underlying changes in hidden state.

In the current study, we develop this idea further using a task-irrelevant visual stimulus (or “impulse stimulus”) to drive a vWM-specific impulse response function that could be measured non-invasively using EEG. Participants performed a two-alternative vWM discrimination task that requires precise maintenance of the orientation of a memory item during a delay interval (Bays and Husain, 2008). Critically, on a subset of trials we presented a fixed high-contrast impulse stimulus designed to drive neural activity in the visual system. We predicted that the evoked response should differentiate the memory condition (i.e., the remembered orientation), even in the absence of vWM-discriminative delay activity.

To anticipate the results, multivariate decoding at posterior electrodes accurately discriminated the orientation of the memory item during stimulus encoding. Consistent with previous evidence for dynamic coding in neural populations (Meyers et al., 2008; Stokes et al., 2013) and scalp-level patterns (Cichy et al., 2011), the discriminative patterns were dynamic during stimulus processing. After the initial dynamic trajectory, discrimination decayed to near-baseline levels during the delay period. Importantly, the impulse stimulus reactivated vWM-specific activity patterns, consistent with the hypothesis that vWM content could be stored in an “activity-silent” neural format. Interestingly, although the impulse response pattern differentiated the vWM-stimulus, the discriminative pattern did not match the patterns during memory encoding. This experiment provides a novel proof-of-concept of a potentially powerful method for inferring hidden neural states.

## Methods

### Participants

Twenty-four healthy adults (12 female, mean age 22.2 years, range 18–38 years) were included in the experiment and analyses. During recruitment, four additional participants were excluded from all analyses due to excessive eye-movements and eye-blinks (more than 20% of trials were contaminated). All participants received a monetary compensation of £10/h and gave written informed consent. The study was approved by the Central University Research Ethics Committee of the University of Oxford.

### Apparatus and stimuli

The experimental stimuli were generated and controlled with the freely available MATLAB extension Psychophysics Toolbox (Brainard, 1997) and presented at a 100 Hz refresh rate and a resolution of 1680 × 1050 on a 22″ Samsung SyncMaster 2233RZ. A USB keyboard was used for response input. The viewing distance was set at 64 cm.

A gray background (RGB = [150 150 150]) was maintained throughout the experiment. Memory items were circular sine-wave gratings presented at a 20% contrast. The memory probes were circular, 100% contrast gratings underlying a square-form function. The radius and spatial frequency was fixed for both types of stimuli (2.88°, and 0.62 cycles per degrees), and the phase was randomized. The memory items' orientations were uniformly distributed, and angle difference between memory item and probe within each trial was uniformly distributed across 20 angle differences (±4°, ±5°, ±7°, ±9°, ±12°, ±15°, ±20°, ±26°, ±34°, ±45°). The impulse item was a high-contrast, black-and-white round “bull's-eye” in the same size and spatial frequency as the memory items and probes. All stimuli were presented centrally. Accuracy feedback was given with high (880 Hz) and low (220 Hz) tones for correct and incorrect responses, respectively.

### Procedure

Participants were seated in a comfortable chair and the keyboard was placed either on their lap or on a table in front of the participants. The participants' task was to memorize the orientation of the presented low-contrast grating and to press the “m” key with the right index finger if the probe was rotated clockwise and the “c” key with the left index finger if the probe was rotated counter-clockwise relative to the previously presented memory item. They were instructed to respond as quickly and as accurately as possible.

**Figure 1:**
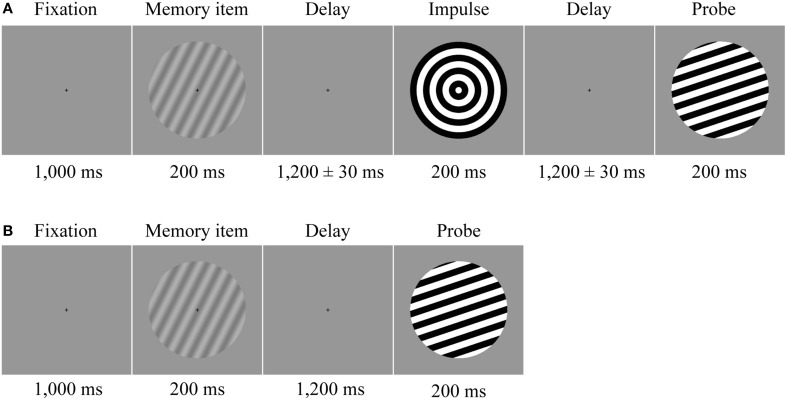
**Trial structure**. Participants memorized the orientation of a low contrast sine-wave grating. **(A)** In half of the trials a neutral impulse stimulus was shown after the initial delay. The onset of the impulse was jittered by ±30 ms. The force-choice discrimination memory probe was presented after a second delay period. **(B)** In the other half of the trials, determined randomly, the probe was presented instead of the impulse after the first delay.

Each trial began with the presentation of a fixation cross, which stayed on the screen until probe presentation. After 1000 ms the memory item was presented for 200 ms. In half of the trials (i.e., “long” trials), the following delay period was 2600 ms, after which the probe was presented for 200 ms. In the delay period at either 1170 (“early-impulse” trials) or 1230 ms (“late-impulse” trials) after the memory item, the impulse stimulus was presented for 200 ms (Figure 1A), which the participants were instructed to ignore. The temporal jitter was introduced to allow us to test whether any effect on stimulus decoding was specifically time-locked to the impulse. In the other half of trials (“short” trials), the response probe was presented 1200 ms after memory item (Figure 1B). These short trials were included to ensure that participants would pay attention throughout the delay period of the long trials. After probe offset, the screen remained blank until response-input. A feedback tone was then played for 100 ms and the next trial automatically began after 500 ms. Every 24 trials a performance summary screen, with the average accuracy and median reaction of all trials thus far, was shown. Participants could use this moment to take short breaks. The trial conditions were randomized across the entire session and participants completed 1600 trials in total (400 early-impulse trials, 400 late-impulse trials, and 800 short trials) over a time period of approximately 165 min (including breaks).

### Behavioral analysis

Memory performance was analyzed with the freely available MATLAB extension MemToolbox (Suchow et al., 2013). The standard mixture model of visual working memory (Zhang and Luck, 2008) was fit separately for each participant (*N* = 24) and trial-length condition. The model assumes that the distribution of response errors has two distinct causes: (1) Pure guesses, which result in a uniform distribution of errors across all angle differences in the forced-choice paradigm. (2) Variability in the precision of the remembered item, which, even though the item is memorized, can result in errors at particularly small angle differences between memory item and probe. Although the main purpose of this analysis was simply to confirm that our participants could reliably memorize the low-contrast memory item in this experiment, for completeness we also performed paired-samples *t*-tests on guess rate and memory variability between trial-length conditions.

### EEG acquisition

The EEG was recorded using NeuroScan SynAmps RT amplifier and Scan 4.5 software (Compumedics NeuroScan, Charlotte, NC) from 61 Ag/AgCl sintered surface electrodes (EasyCap, Herrsching, Germany) laid out according the to the extended international 10–20 system (Sharbrough et al., 1991) at 1000 Hz sampling rate. The anterior midline frontal electrode (AFz) was reserved as the ground. Electrooculography (EOG) was recorded from electrodes placed below and above the right eye and from electrodes placed to the left of the left eye and to the right of the right eye. Impedances were kept below 5 kΩ. Data were filtered online using a 200 Hz low-pass filter and the electrodes were referenced to the right mastoid.

### EEG preprocessing

Offline, the signal was re-referenced to the average of both mastoids, down-sampled to 250 Hz with 16-bit precision and band pass filtered (0.1 Hz high-pass and 40 Hz low-pass) using EEGLAB (Delorme and Makeig, 2004). Because we were only interested in posterior electrodes for this study, re-referencing to global average could unnecessarily introduce additional noise from frontal channels. Nevertheless, for completeness, we confirmed that the results are qualitatively similar using both reference schemes. The data were then epoched from −200 to 1400 ms relative to the onset of the memory item for the short, no-impulse trials, and from −200 to 2800 ms for the long, impulse trials. Both long and short epochs were then baseline-corrected using the 200 ms prior to memory item onset. Subsequent artifact detection and trial rejection was performed via visual inspection and focused exclusively on the EOG channels and the 17 posterior channels of interest included in the analyses (P7, P5, P3, P1, Pz, P2, P4, P6, P8, PO7, PO3, POz, PO4, PO8, O1, Oz, O2). Trials containing saccadic eye-movements at any point in time, blinks during stimulus presentation, or other non-stereotyped artifacts were rejected from all further analyses. Impulse trials were subsequently re-epoched to two shorter epochs, time-locked to the memory item (−200 to 1400 ms) or to the impulse stimulus (−200 to 1400 ms). Finally, the data were smoothed with a Gaussian kernel (*SD* = 8 ms).

### EEG analysis

#### Multivariate pattern analysis

To determine whether the pattern of the EEG signal across the posterior channels of interest contained information about the remembered item, we used the Mahalanobis distance (Mahalanobis, 1936; De Maesschalck et al., 2000) to perform pair-wise comparisons between sets of trials in which orthogonal orientations were presented.

**Figure 2:**
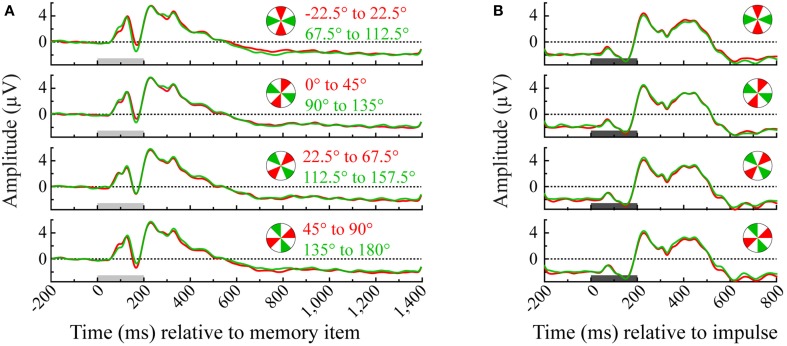
**Event-related potentials of each angle bin averaged over the occipital channels (O1, Oz, and O2)**. Illustrated are all pairwise orthogonal angle bin comparisons that were made in the multivariate analysis of the memory item epoch **(A)** and impulse epoch **(B)**. Light-gray and dark-gray bars represent the presentation of memory item and impulse stimuli, respectively.

Trials were divided across four angle bins two times and only orthogonal angle bins were compared in the multivariate analysis (0° to 45° vs. 90° to 135°; 45° to 90° vs. 135° to 180°; −22.5° to 22.5° vs. 67.5° to 112.5° and 22.5° to 67.5° vs. 112.5° to 157.5°). For illustration, see Figure 2 for the event-related potentials of occipital electrodes (O1, Oz, and O2) for each pairwise comparison between orthogonal angle-bins.

**Figure 3:**
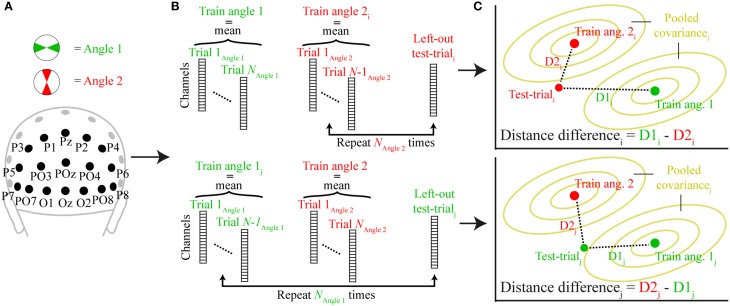
**A schematic representation of the trial-wise Mahalanobis distance analysis**. **(A)** The signal for two orthogonal angle bins (angle 1 and angle 2) was extracted from all posterior channels at a specific time point. **(B)** A single trial was either removed from angle 2 (top; test-trial_i_) or angle 1 (bottom; test-trial_j_) and the mean signal for each angle condition of all other trials comprised the training set (train angle 1, train angle 2). **(C)** The Mahalanobis distances of the left-out test-trial to train angle 1 (D1) and train angle 2 (D2) illustrated in two-dimensional space. The pooled covariance is computed from the training data. When the test trial belongs to angle bin 2, D2_i_ is subtracted from D1_i_ (top), when it belongs to angle bin 2, D1_j_ is subtracted from D2_j_ (bottom). This procedure is repeated for each trial and time-point and the resulting distance differences are averaged across all trials.

We used a leave-one-trial-out cross-validation approach to calculate, on each trial, the multivariate dissimilarity (Mahalanobis distance) of that trial to the average of all other trials in the same angle bin, relative to the dissimilarity of that trial to the average of all trials in the orthogonal angle bin. Mahalanobis distances of the test trial were computed for each time point as follows:

$$\begin{array}{l} D1\;=\sqrt{\begin{array}{r} {\left(\text{Train angle }1-\text{ Test trial}\right)}^{T}*{\text{pC}}^{+}*\\ \left(\text{Train angle }1-\text{ Test trial}\right) \end{array}\;}\\ D2=\sqrt{\begin{array}{r} {\left(\text{Train angle }2\;-\text{ Test trial}\right)}^{T}*{\text{pC}}^{+}*\\ \left(\text{Train angle }2\;-\text{ Test trial}\right) \end{array}} \end{array}$$

where “Train angle 1” and “Train angle 2” are row vectors containing the average signals of angle bins 1 and 2 (excluding the test trial) of each channel, and “pC^+^” is the pseudo inverse of the error covariance matrix. The error covariance was estimated by pooling over the covariances of each angle condition, estimated from all trials within each condition (excluding the test trial) using a shrinkage estimator that is more robust than the sample covariance for data sets with many variables and/or few observations (Ledoit and Wolf, 2004; Kriegeskorte et al., 2006). The variables “Train angle 1,” “Train angle 2,” and “pC^+^” are all part of the training set, on which “Test trial,” a row vector containing the signal of each channel of the left-out test-trial, is tested on. This was done by computing the difference between the two Mahalanobis distances between “Test trial” and “Train angle 1” (D1) and “Test trial” and “Train angle 2” (D2). The same-angle bin distance was always subtracted from the orthogonal-angle bin difference (so if the “Test trial” was part of angle bin 1 then D1 would be subtracted from D2). If the signal indeed contained information about the memory item at that time point, this distance difference should be positive (because the orthogonal-angle bin distance should be higher than the same-angle bin distance). See Figure 3 for a schematic overview of the analysis. This procedure was performed for all trials and all previously defined angle bin comparisons, resulting in two equivalent estimates of distance differences per trial. Observed distances were then averaged over the two estimates, and across trials, to derive a single value for each time point and each participant for subsequent statistical testing and plotting.

#### Cross-temporal analysis

To explore the dynamics of information processing, and to test if the informative signal cross-generalizes to other time points (King and Dehaene, 2014), we computed a cross-temporal extension of the Mahalanobis analysis described above. The difference between condition-specific distances was computed as described above. However, instead of training and testing only on the same equivalent time points, train/test sliding windows were decoupled: The training data consisting of “Train angle 1,” “Train angle 2” and the corresponding pseudo inverse of the covariance matrix (as described above) at train time *Y* was used to compute the distances to the test-trial at test time *X* (e.g., Stokes et al., 2013). After computing the distance differences for all possible train-test time combinations and averaging across all test trials, the results were combined into a cross-temporal matrix in which differences along the diagonal correspond directly to the time-resolved analyses already discussed, but off-diagonal coordinates reflect the extent to which the underlying discriminative neural patterns cross-generalize between train-test time points. This cross-temporal analysis was carried out within each trial epoch separately (memory-item and impulse), as well as across epochs, where the train data was taken from the impulse epoch and tested on all trials within the memory item epoch and vice versa, resulting in four cross-temporal discrimination matrices.

#### Univariate analysis

To explore to what extent the differences in the EEG signal between memory items is driven by amplitude rather than pattern differences, we performed the univariate equivalent to the multivariate analysis described above. Instead of calculating the difference between the same- and orthogonal-angle bin Mahalanobis distances, the difference between the absolute same- and orthogonal-angle bin voltage differences averaged across all 17 posterior channels was computed.

#### Significance testing

Statistics of one-dimensional EEG-analyses were inferred non-parametrically (Maris and Oostenveld, 2007) with sign-permutation tests. For each time-point, the decoding value of each participant was randomly multiplied by 1 or −1. The resulting distribution was used to calculate the *p*-value of the null-hypothesis that the mean discrimination-value was equal to 0. Cluster-based permutation tests were then used to correct for multiple comparisons across time using 10,000 permutations, with a cluster-forming threshold of *p* < 0.01. The significance threshold was set at *p* < 0.05 and all tests were two-sided. Significance tests were carried out separately for the memory item (0–1400 ms) and the impulse (0–800 ms). The sample size of all tests was 24.

### Data sharing

In accordance with the principles of open evaluation in science (Walther and van den Bosch, 2012), all data and fully annotated analysis scripts from this study are publicly available at http://datasharedrive.blogspot.co.uk/2015/05/revealing-hidden-states-in-working.html.

We also hope these data and analyses will provide a valuable resource for future re-use by other researchers. In line with the OECD Principles and Guidelines for Access to Research Data from Public Funding (Pilat and Fukasaku, 2007), we have made every effort to provide all necessary task/condition information within a self-contained format to maximize the re-use potential of our data. We also provide fully annotated analysis scripts that were used in this paper. Any further queries can be addressed to the corresponding author.

## Results

### Behavioral results

**Figure 4:**
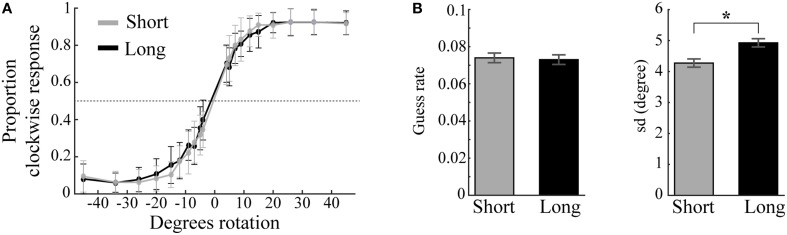
**Behavioral performance and model parameters**. **(A)** Mean proportion of clockwise responses as a function of angle difference between memory item and probe plotted separately for short (gray) and long (black) trials. Error bars are standard deviations. **(B)** Guess rates and memory variability (sd) for short and long trials estimated by the standard mixture model of visual working memory (Zhang and Luck, 2008). Long trials result in significantly higher sd than short trials (^*^). Error bars are normalized standard errors.

Visual working memory performance (Figure 4A) was modeled separately for short and long trials, each consisting of 800 trials. The difference in guess rates for short (*M* = 0.074, *SD* = 0.048) and long trials (*M* = 0.073, *SD* = 0.047) was not statistically different [*t*_(23)_ = 0.182, *p* = 0.858]. On the other hand, the standard deviation of remembered items (sd) was significantly different between trial length conditions [*t*_(23)_ = 2.458, *p* = 0.022]: sd was lower for short trials (*M* = 4.272, *SD* = 1.318) than for long trials (*M* = 4.927, *SD* = 1.292; Figure 4B). Whether this decrease in precision in long trials is due to the increase in trial duration (Zhang and Luck, 2009) or the possible interference effect of the impulse stimulus (Magnussen et al., 1991) cannot be concluded, as the present study was not designed to address this issue.

The very low guess rates in both conditions provided evidence that the participants had little difficulty to reliably memorize the low contrast angle stimuli. Because most errors were attributed to noise in mnemonic precision rather than absolute forgetting, we included both incorrect and correct trials in all EEG analyses.

### Memory item discrimination during and after item presentation

**Figure 5:**
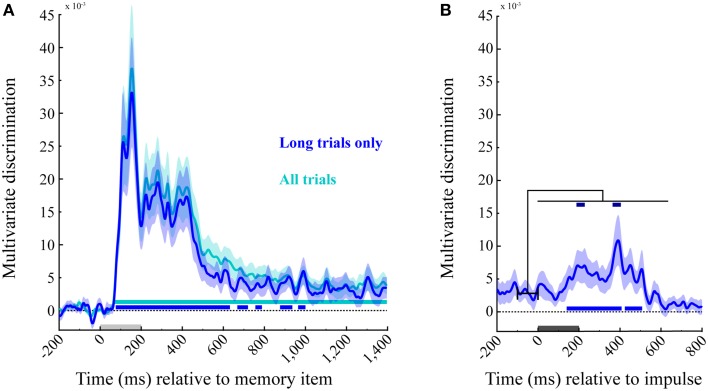
**Multivariate discrimination of the memory item across time**. **(A)** Memory item epoch. The discrimination for both trial types (in cyan), and exclusively for the long trials used in the impulse response analysis (in blue). Significant positive clusters are marked with bars in the corresponding colors. **(B)** Impulse epoch. The discrimination of memory item is shown for long trials (in blue), with positive clusters are marked in the corresponding significance bar along the bottom. Significant increases in discrimination compared to the mean discrimination 100 ms prior to impulse onset are indicated with dark-blue bars at the top. Light-gray and dark-gray bars represent memory item and impulse presentation, respectively. Error bars are standard deviations from the permuted null-distributions.

The averaged trial-wise difference in Mahalanobis distances between across- and within-angle conditions enabled us to decode the memory items from the EEG signal of the posterior channels as a function of time. A statistically significant cluster emerged 68 ms after memory item onset, and lasted until the end of this epoch (1400 ms, cluster *p* < 0.001; Figure 5A, cyan). Because the impulse analysis was only based on 50% of trials, we also analyzed the memory encoding effect only on corresponding long trials (Figure 5A, blue), enabling a power-matched comparison between the memory item- and impulse-epoch. This revealed several significant decoding clusters: 76–632 ms (*p* < 0.001), 668–720 ms (*p* = 0.023), 756–788 ms (*p* = 0.047), 876–936 ms (*p* = 0.016), and 964–1000 ms (*p* = 0.036).

### Memory item discrimination during and after impulse presentation

The same analysis as above was performed on the subsequent epoch for long trials, time-locked to the impulse onset. Significant temporal clusters of above-chance discrimination were detected at 140–408 ms (*p* < 0.001) and 424–508 ms (*p* = 0.005 after impulse onset (Figure 5B, blue, bottom).

### Decoding accuracy increases significantly after impulse presentation

Since the decoding accuracy does not seem to drop completely to chance levels in the initial delay period, we also tested whether the presentation of the impulse results in a significant *increase* in discriminability. To this end, we subtracted the mean discriminability between −100 and 0 ms prior to impulse onset from the discrimination values after impulse onset. Two significant clusters were identified: 188–232 ms (*p* = 0.012) and 364–0.404 ms (*p* = 0.016). These results confirm that discrimination accuracy increased significantly after impulse presentation (Figure 5B, blue, top).

### The memory item and impulse show dynamic coding

**Figure 6:**
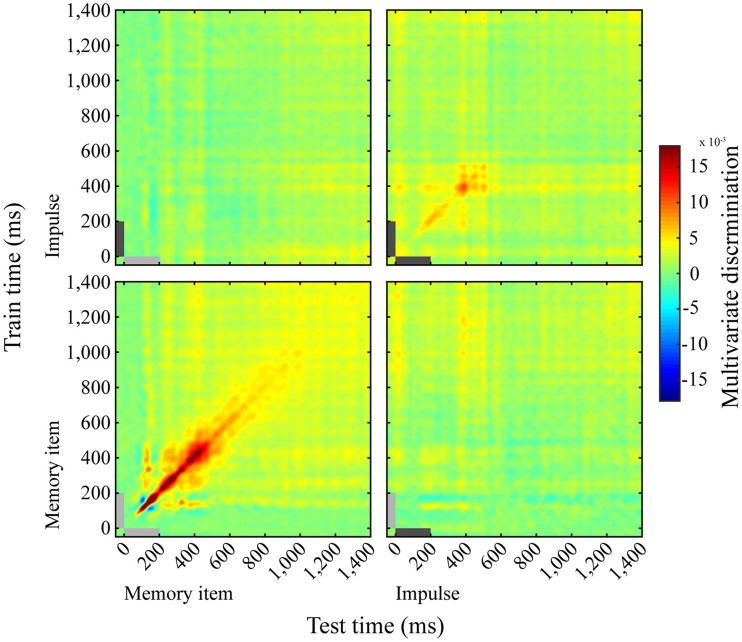
**Dynamics of memory item discrimination**. Mean discrimination matrices derived from training and testing on all time-point combinations. Light-gray and dark-gray bars represent memory item and impulse presentation, respectively.

The cross-temporal analysis of the memory item epoch using both long and short trials showed a dynamic coding pattern. Discrimination was greatest when trained and tested on the same time-points, as opposed to different time-points (Figure 6, lower left). The impulse response, though weaker than the memory item response, suggested a dynamic coding pattern as well (Figure 6, upper right).

### Memory item and impulse coding do not cross-generalize

We saw no evidence for cross- generalization between the neural patterns evoked by the memory stimulus and the impulse response, either when the training set was taken from the impulse epoch and tested on the memory item epoch (Figure 6, top left), or the other way around (Figure 6, bottom right).

### Discrimination accuracy is time-locked to impulse onset

The increased discrimination accuracy shortly after the impulse could in principle be explained by a probe expectancy effect. Because the memory probe is presented on half the trials at this point, participants might prepare to respond to the probe. This could result in a more “active” maintenance of the memory item (e.g., Watanabe and Funahashi, 2007), which in turn could improve decoding accuracy. Although we do not find any evidence for a progressive ramp-up in discriminability at this time, this does not rule out a very precise form of temporal expectation.

To address this potential issue directly, we had introduced a very subtle temporal variability in the presentation of the impulse stimulus. Our reasoning was as follows: If discriminability is tightly time-locked to the variable onset of the impulse, rather than to the expected onset of the probe relative to the memory item, we can sensibly attribute the observed boost in discriminability to the presentation of the impulse stimulus.

**Figure 7:**
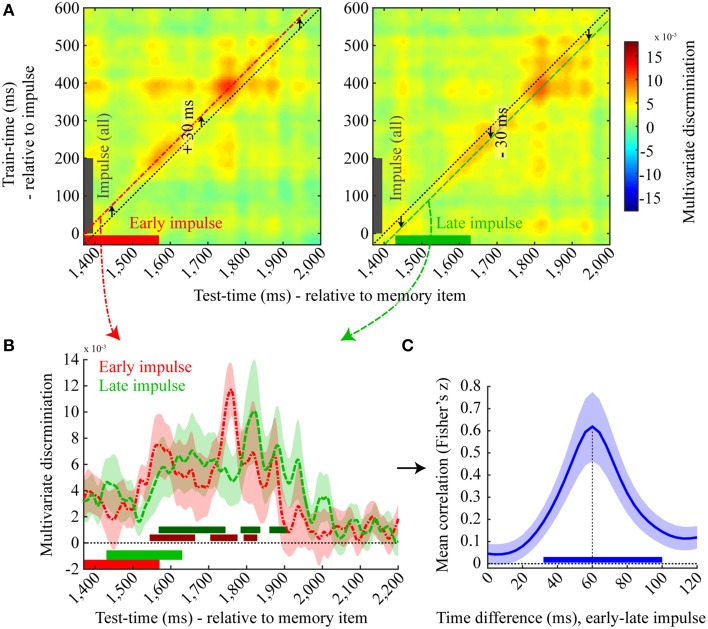
**Effect of late impulse onset**. **(A)** Mean discrimination matrices derived from training on all impulse trials, time-locked to impulse onset, and testing separately on early (left, red) and late (right, green) impulse onset trials. The black dotted lines illustrate the multivariate discrimination when tested on the average impulse onset relative to memory item (1400 ms) but trained relative to the actual impulse onset (0 ms). Discrimination for early onset trials is highest when the training time is shifted by +30 ms (left, red line) and highest for late onset trials when shifted by −30 ms (right, green line). **(B)** A one dimensional plot of the early (red) and late (green) onset discriminations trained at +30 ms and −30 ms relative to impulse onset, respectively. Significant positive clusters of each onset condition are indicated by bars in a darker shade of the corresponding colors. Error bars are standard deviations of the permuted null distributions. **(C)** Mean correlations (Fisher's z) between the decoding time-course for the early and late impulse onset trials as a function of different temporal shifts. Mean correlation peaks at 60 ms. The blue bar illustrates the significant positive cluster of correlations. Error bars are standard deviations of the permuted null distributions.

We therefore plotted the cross-temporal matrices of the discrimination of the early and late impulse onset trials separately (Figure 7A) time-locked to memory item onset, where the training data of both matrices was based on all impulse trials time-locked to impulse onset. As is apparent from the figure, the highest discrimination effect is not along the diagonal (where the test and train times correspond to the mean impulse onset and the actual impulse onset of all trials, respectively). Rather, for the early impulse trials, discrimination is highest when the training time is shifted by +30 ms, while a −30 ms shift is best for the late impulse trials. We then plotted and analyzed the discriminations of the early and late impulse trials based on these shifted training times (Figure 7B). Three positive significant clusters were found both in the early-onset condition (1544–1664 ms, *p* = 0.003; 1704–1776 ms, *p* = 0.007; 1792–1828 ms, *p* = 0.028) and in the late-onset condition (1568–1744 ms, *p* < 0.001; 1784–1836 ms, *p* = 0.012; 1860–1908 ms, *p* = 0.016). As is apparent from both the figure and the significant clusters, the time course of the late impulse onset trials is clearly later than the early onset trials.

To more directly test for the expected 60 ms latency shift in discrimination accuracy corresponding to the onset difference of the two impulse stimuli, we computed the Pearson's correlation between discrimination values of the time window from 1370 to 2170 ms of the early impulse onset condition with different time windows of the same length of the decoding values of the late impulse onset condition. Correlation coefficients were computed between the same time windows (0 ms difference) as well as for each 4 ms step up to a difference of 120 ms, resulting in 31 correlation values for each participant in total (Figure 7C). The mean correlation clearly peaked at a 60 ms difference and a cluster-corrected permutation test on the Fisher transformed correlation values showed that only the correlation coefficients between a time-difference of 32 to 100 ms were significantly positive across subjects (*p* < 0.001). These results provide clear evidence that the decoding time-course was time-locked to the onset of the impulse.

### Memory item discrimination is not simply driven by mean amplitude difference

**Figure 8:**
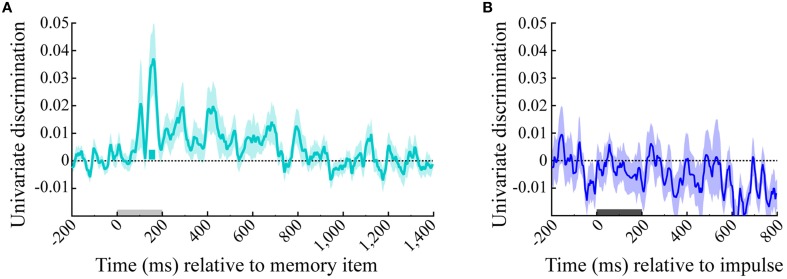
**Univariate discrimination of the memory item**. The cyan and blue lines show the univariate discrimination of the memory item of the **(A)** memory item and **(B)** impulse epoch, respectively. The cyan bar indicates the significantly positive discrimination cluster of the memory item epoch. Light-gray and dark-gray bars represent memory item and impulse presentation, respectively. Error bars are standard deviations of the permuted null-distributions.

The univariate analysis that was based on the averaged signal of all posterior electrodes showed significant memory item discrimination only shortly after memory item onset, where a single short significant cluster was present (140–168 ms, *p* = 0.022). No significant discrimination could be made within the impulse epoch (Figure 8).

## Discussion

We report the results of a novel method to recover visual working memory states that are otherwise hidden to EEG using a functional perturbation approach. We presented a high-energy visual impulse stimulus during the vWM delay period and measured the visual evoked response. Critically, we found that the impulse response carried significant information about the contents in vWM. Using multivariate analysis, we could decode the orientation of the previous memory item from the impulse-driven visual response. This provides important proof-of-principle evidence for the feasibility of exploring hidden neural states with non-invasive EEG, with important implications for working memory (Stokes, 2015).

We used Mahalanobis distances to compute the multivariate dissimilarity between the evoked response during maintenance of specific orientations. The Mahalanobis distance is superior to Euclidean distance (Stokes et al., 2013) because it accounts for the covariance structure of the noise between features (Kriegeskorte et al., 2006). In the current study, features were EEG sensors, which are known to be highly correlated. Analysis of the evoked response to the memory stimulus clearly validated this multivariate method as a powerful approach for decoding task-relevant parametric dimensions. Robust orientation discrimination was observed in the EEG activity as early as 68 ms after the presentation of the memory stimulus. Decoding peaked at around 160 ms, before decaying into the memory delay period. Despite returning almost to baseline prior to the onset of the impulse stimulus, we observed a robust “reactivation” in decodability of the memory item that peaked at 200 and 360 ms after the impulse stimulus.

The impulse onset was temporally jittered by ±30 ms. The rationale for introducing this variability was to control for the possibility that reactivation could be explained by temporal expectation. On half the trials, the response probe was presented instead of the impulse stimulus. This was to ensure that participants were attending throughout the delay period. However, previous studies have shown that temporal expectation can also result in a ramp-up of item-specific delay activity (Takeda and Funahashi, 2004; Watanabe et al., 2009; Barak et al., 2010). Ramp-up activity could reflect a build-up of temporal expectation (Nobre et al., 2007), which could trigger attention-related pre-activation of the task-relevant template, as previously observed in monkey PFC (Rainer et al., 1999) and the human visual system (Stokes et al., 2009). Jittering the impulse onset time allowed us to differentiate the relative contribution of temporal expectation and of the impulse response. This subtle temporal offset allowed us to test whether reactivation was indeed time-locked to the impulse stimulus, or whether decodability was better explained by the temporal structure of the task.

Visual inspection of the decodability time-course locked to the impulse probe already suggests that temporal expectation is not a plausible account. It would be surprising if template-reactivation could be so precise over an interval as long as 1.2 s. Moreover, plotting the impulse response for the different impulse onset times relative to the onset of the memory stimulus provides an estimate of the time-locking to the stimulus onset (Figure 7B). As expected, the decodability profiles appear offset by approximately 60 ms. Finally, a correlation analysis of the decodability time-courses between impulse onsets confirmed that the correlation peaked at an offset of 60 ms. Overall, this pattern of results is consistent with the prediction that a neutral stimulus presented during the delay period drives activity in the memory network, resulting in a patterned response that systematically reflects the representational characteristics of the information in working memory (i.e., orientation).

Previous studies have argued that early visual cortex is important for vWM (Pasternak and Greenlee, 2005). For example, Harrison and Tong conducted an fMRI study using a very similar paradigm as the current design (Harrison and Tong, 2009). Using multivariate analyses, they found significant decoding during the delay period despite an absence of above-baseline activity levels. This suggests that subtle activity patterns in fMRI could also reflect hidden states (patterned spontaneous activity). Computational modeling provides evidence that spontaneous spiking activity should be patterned by the hidden state (Sugase-Miyamoto et al., 2008). Moreover, we previously found evidence for significant pattern separation in monkey PFC, despite activity levels that were no greater than the pre-trial baseline (Stokes et al., 2013). Increasing the overall level of activity increased the pattern separation in that study. Future research could explore the relationship between spontaneous activity patterns measured with fMRI, single unit recording, and EEG.

It is also possible that the activity observed by Harrison and Tong (2009) actually reflected attentional preparation (Stokes et al., 2009) or imagery-related activity (Stokes et al., 2011; Albers et al., 2013). Indeed, it is almost impossible to separate potential non-working memory contributions in their design (Stokes, 2011). In the current study, we clearly dissociate impulse-driven decoding from temporal expectation. Moreover, visual imagery is unlikely to be triggered so rapidly by the impulse stimulus. It would be important for future research to explore the relationship between discriminating stimulus-driven and non-driven activity as a function of attention and imagery to further pinpoint the relative contribution of different neural states to these separable, but interrelated cognitive functions.

We also observed evidence for dynamic coding of the memory stimulus. Cross-temporal analyses clearly revealed superior discrimination along the diagonal axis, reflecting within-time generalization, relative to off-diagonal coordinates representing cross-temporal generalization. This is the hallmark pattern for dynamic coding, indicating that the discriminative patterns vary over time (King and Dehaene, 2014). Previously, Cichy and colleagues observed a similar pattern in MEG data during perceptual categorization (Cichy et al., 2011), consistent with similar results from intracranial recordings in monkey visual (IT; Meyers et al., 2008), parietal (Crowe et al., 2010) and prefrontal cortices (Meyers et al., 2008; Stokes et al., 2013). There was also some evidence for a dynamic coding pattern in the impulse response, suggesting that the impulse response might be best conceptualized as a memory-specific trajectory, although future research would need to clarify this interpretation.

Interestingly, we found no evidence for cross-generalization between the neural patterns evoked by the memory stimulus and the impulse response. Again, this could be interpreted as an extension of dynamic coding. The same task parameters are represented in both epochs (i.e., memory orientation), but using independent coding schemes. Epoch-independent coding schemes could be optimal for structured high-level representations (Sigala et al., 2008). However, this result could also reflect a fundamental difference in patterns of activity that modulate hidden states, and the patterns of activity that are emitted from a particular impulse stimulus. Indeed, the current results are consistent with the hypothesis that the impulse response should be an interaction between the input pattern and the current hidden state, rather than a simple “reactivation.” Readout of the hidden state from the EEG response only requires a systematic relationship between the impulse response and the hidden state. By contrast, downstream cortical areas that read out the hidden state to generate a response might need to learn how to decode a time- and context-varying hidden state to access a memorized orientation. Recent theoretical models have shown that unsupervised read-out of dynamically changing states is in principle possible (Sussillo and Abbott, 2009; Sussillo, 2014).

Although this proof-of-principle experiment does not provide the definitive test for “activity-silent” working memory, the results are nonetheless consistent with a number of key predictions. First, memory-discriminative information effectively returns to baseline after initial encoding. Although this is essentially a null effect, the decay function is consistent with studies decoupling persistent content-specific delay activity and memory-guided behavior (Sreenivasan et al., 2014). Secondly, impulse-driven reactivation is consistent with a context-dependent response of a memory-configured hidden state (Mongillo et al., 2008; Sugase-Miyamoto et al., 2008). Finally, the dynamic trajectory during memory encoding is also consistent with a more general dynamic coding framework for working memory (Stokes, 2015).

Irrespective of any particular theoretical framework, the current experiment also provides an important demonstration of combining a functional perturbation approach with multivariate decoding to reveal otherwise hidden neural states. Activity states that we usually measure with non-invasive recordings only provide an incomplete picture of the diversity of neural states underlying cognition. This might be especially true for more tonic cognitive states, such as working memory, attention, or task set. Activity-silent representations pose an obvious problem for contemporary neuroscience, which is dominated by measurement and analysis of activity states. The ultimate success of future research will depend on new approaches to existing measurement techniques to probe diverse neural states, including “activity-silent” states. We believe that this paper provides an important proof-of-principle toward an accessible non-invasive approach. Non-invasive brain stimulation could be used in combination with EEG to probe hidden states (Bortoletto et al., 2015). The advantage of transcranial magnetic stimulation is that the response profile of distinct brain networks can be targeted specifically (Rosanova et al., 2009), but with the major disadvantage that the stimulation artifact effectively precludes analysis of the initial local response to the perturbation. While this is less problematic for measuring context-dependent changes in effective connectivity between distant brain areas (Taylor et al., 2007), this limitation could easily obscure the kind of effect studied here.

In conclusion, we provide useful proof-of-principle demonstration of the utility of combining a functional perturbation approach with EEG to reveal otherwise silent neural states. Although these results are consistent with a dynamic coding framework that suggests visual working memory could be encoded in an “activity-silent” state, the main purpose of the experiment was to develop a powerful tool for exploring cognitive states that cannot otherwise be differentiated with EEG. Future experiments will be able to exploit this novel approach in more complex experimental designs to tease apart the key coding principles underlying visual working memory.

### Conflict of interest statement

The authors declare that the research was conducted in the absence of any commercial or financial relationships that could be construed as a potential conflict of interest.
